# Older Patients with Acute Myeloid Leukemia Deserve Individualized Treatment

**DOI:** 10.1007/s11912-022-01299-9

**Published:** 2022-06-02

**Authors:** David C. de Leeuw, Gert J. Ossenkoppele, Jeroen J. W. M. Janssen

**Affiliations:** Department of Hematology, Amsterdam University Medical Centers, Location VUmc, De Boelelaan 1117, 1081 HV Amsterdam, The Netherlands

**Keywords:** Acute myeloid leukemia, Elderly, Fitness, Treatment, Intensive chemotherapy, Hematopoietic stem cell transplantation, Hypomethylating agents, Targeted therapy, FTL3 inhibitors, IDH inhibitors, Venetoclax, Enasidenib, Ivosidenib, Gilteritinib, Midostaurin, Gemutuzumab ozogamicin, Glasdegib, CPX-351, Vyxeos, CC-486, Azacitidine, Decitabine

## Abstract

**Abstract:**

**Purpose of Review:**

Treatment of elderly patients with acute myeloid leukemia is a known challenge for hematologists due to patient diversity, heterogeneous disease biology, and a rapidly evolving treatment landscape. Here, we highlight the importance of determining fitness, review the latest therapeutic developments, and discuss clinical scenarios to provide guidance on individualized treatment for older AML patients.

**Recent Findings:**

Several factors, like age, performance status, and comorbidities, play a role in fitness and are associated with outcome. Comorbidity scoring systems and geriatric assessments are tools to help physicians select the most appropriate treatment for each patient. The addition of venetoclax, targeted therapy with IDH1/2 and FLT3 inhibitors, and enhanced formulas of existing drugs like CPX-351 and oral azacitidine have improved responses and outcomes.

**Summary:**

New drugs and combination therapies have increased the therapeutic options for elderly AML patients but determination of fitness and disease biology is essential to select patient-tailored treatments.

## Introduction

Acute myeloid leukemia (AML) is primarily a disease of the elderly with a median reported age at diagnosis of around 70 years [1]. It is estimated that in 2021, over 12,000 patients ≥ 65 years have been diagnosed with AML in the USA, and due to the aging population, this number will rise considerably in the next decades [2].

In contrast to younger patients, whose 5-year overall survival rates improved significantly since the 1970s (from 13 to 55%), survival in elderly patients remains poor with only slight improvements (from 8 to 17%) [3]. Several factors underlie this difference. For one, significant advances in allogeneic hematopoietic stem cell transplantation (HSCT) and optimized (support during) intensive chemotherapy (IC), that have improved outcomes in younger patients, are unfeasible in most elderly patients. Higher age comes with decreased performance status (PS) and the prevalence and severity of conditions that complicate intensive treatment, such as cardio-pulmonary disease, renal disease, and dementia, increase across cancer patients’ age spectrum [4]. Moreover, compared to younger patients, elderly patients more often present with moderate to severe comorbidities and irreversible end-organ disease which is associated with inferior overall survival (OS) in patients receiving IC for AML [4, 5].

Next to the differences in host factors between younger and elderly AML, there is a difference in disease biology. Elderly AML patients more often present with unfavorable cytogenetics or molecular abnormalities and a greater proportion has therapy-related (tAML) or secondary AML (sAML) than younger patients [6–8]. Frequently mutated genes include mutations in* TP53 *and chromatin–spliceosome genes, such as *SRSF2* and *ASXL1*, which are independently and additively associated with a poor outcome [9]. In contrast, frequencies of favorable cytogenetics and mutations, like *NPM1*, are markedly lower in elderly AML [6, 10].

Together, host factors that limit the ability to receive IC and difficult-to-treat disease biology contribute to poorer outcome in elderly patients with AML. However, while this patient population is heterogeneous, elderly patients in general benefit from IC when deemed fit [11]. Registry data showed that more than 60% of patients aged 70–74 were considered fit for IC, and that this was still 45% in patients between 75 and 79 years of age [11]. For older, medically non-fit patients, hypomethylating agents (HMAs) are a relatively non-intensive treatment option which modestly improves survival rates compared to best supportive care (BSC) [12], but they must be considered a palliative option. Adequate assessment of fitness and characterizing the intrinsic properties of the disease is, therefore, crucial to direct therapy decision-making in older patients with AML.

Recently, the addition of venetoclax to HMA was shown to strongly improve outcome for older, medically non-fit AML patients, to such an extent that choosing the optimal treatment has become challenging [13••]. With the advent of eight other newly FDA-approved drugs for treatment of AML, the isocitrate dehydrogenase (IDH) inhibitors Ivosidenib and Enasidenib, the FMS-like tyrosine kinase 3 (FLT3) inhibitors Midostaurin and Gilteritinib, the anti-CD33 monoclonal antibody gemtuzumab ozogamicin (GO), CPX-351, the hedgehog signaling pathway inhibitor Glasdegib, and the oral HMA CC-486, the answer to the question how to treat elderly patients has become more complicated (Table 1).

**Table 1:** Approved therapies for the treatment of AML. The table shows the various therapies that are approved for the treatment of AML, type of treatment, approved indication, and several advantages and disadvantages. *AML-MRC*, AML with myelodysplasia-related changes; *APL, *acute promyelocytic leukemia*; AXL*, *AXL* receptor tyrosine kinase UFO; *CBF*, core-binding factor; *EMA*, European Medicines Agency*; *FDA, Food and Drug Administration; *FLT3m*, FMS-like tyrosine kinase mutated; *tAML*, therapy-related AML; *HMA*, hypomethylating agent; *IC, *intensive chemotherapy*; IDH1m*, isocitrate dehydrogenase 1 mutated; *IDH2m*, isocitrate dehydrogenase 2 mutated; *LDAC*, low-dose cytarabine; *ND, *newly diagnosed*; NPM1,* nucleophosmin-1; *R/R*, relapsed/refractory; *TKI*, tyrosine kinase inhibitor

Treatment	Type	Approved indication	Advantages (+)/disadvantages (-)
Intensive chemotherapy (7+3)	“Classical” combination of cytarabine (nucleoside metabolic inhibitor) and daunorubicin (anthracycline)	FDA: remission induction in acute non-lymphocytic leukemia of adults and pediatric patients. EMA: remission induction in acute non-lymphocytic leukemia of adults and pediatric patients.	+ Extensive experience + Generally high response rates + Inexpensive – Not suitable for unfit patients due to toxicity
CPX-351 (VYXEOS)	Liposomal formulation of cytarabine and daunorubicine	FDA: adults with ND tAML or AML-MRC. EMA: adults with ND tAML or AML-MRC.	+ Improved outcome compared to IC in tAML/AML-MRC + Real world data shows less toxicity compared to IC + Easier dosing schedule – Prolonged myelosuppression compared to IC (around 7–10 days) – Expensive
Gemtuzumab ozogamicine (MYLOTARG)	Humanized anti-CD33 monoclonal antibody conjugated with calicheamicin	FDA: treatment of ND CD33-positive AML in adults and treatment of R/R CD33-positive AML in adults and in pediatric patients 2 years and older. EMA: combination therapy with daunorubicin and cytarabine for the treatment of patients age 15 years and above with previously untreated, de novo CD33-positive AML, except APL.	+ Survival benefit for patients < 70 years, with *de novo* NPM1m/FLT3wt AML, low-/intermediate-risk karyotypes, and CBF AML – Increased toxicity, especially in older patients
Midostaurin (RYDAPT)	First-generation small molecule that inhibits multiple receptor tyrosine kinases among others FLT3-ITD and TKD (1st-generation inhibitor)	FDA: ND FLT3 mutated AML in combination with standard cytarabine and daunorubicin induction and cytarabine consolidation. EMA: in combination with standard daunorubicin and cytarabine induction and high dose cytarabine consolidation chemotherapy, and for patients in CR followed by midostaurin single-agent maintenance therapy, for adult patients with ND FLT3 mutated AML.	+ Improves outcome combined with IC and maintenance in FLT3 mutated AML compared to IC – Gastrointestinal toxicity
Gilteritinib (XOSPATA)	Second-generation small-molecule FLT3/AXL tyrosine kinase inhibitor	FDA: adult patients with R/R FLT3 mutated AML. EMA: monotherapy for adult patients with R/R FLT3 mutated AML.	+ Improves outcome and response compared to standard salvage treatment in R/R FLT3 mutated AML + More potent than first-generation TKI and generally less side effects + Oral formulation – Associated with differentiation syndrome
Ivosidenib (TIBSOVO)	Small molecule inhibitor of mutant IDH1	FDA: adult ND IDH1 mutated AML who are ≥ 75 years old or who have comorbidities that preclude use of IC induction and adult patients with R/R IDH1 mutated AML. EMA: not approved.	+ Improved response and survival rate when combined with azacitidine in IDH1 mutated ND AML – Associated with differentiation syndrome
Enasidenib (IDHIFA)	Small molecule inhibitor of mutant IDH2	FDA: adult patients with R/R IDH2 mutated AML. EMA: not approved.	+ Improved response rate when combined with azacitidine in IDH2 mutated AML but no demonstrated survival benefit – Associated with differentiation syndrome
Glasdegib (DAURISMO)	Small molecule inhibitor of Hedgehog signaling pathway	FDA: in combination with low-dose cytarabine, for the treatment of ND AML in adult patients who are ≥ 75 years old or who have comorbidities that preclude use of IC induction. EMA: in combination with a low-dose cytarabine when the patient cannot be treated with standard chemotherapy.	+ Improved response and survival rate when combined with LDAC compared to LDAC alone – Response and outcome not better than treatment with HMA only – LDAC is not a preferred treatment in most countries
Venetoclax (VENCLYXTO)	Highly selective small molecule BH3 mimetic inhibiting BCL-2 and BCL-XL	FDA: in combination with HMA or low-dose cytarabine for the treatment of ND AML in adults who are age 75 years or older, or who have comorbidities that preclude use of IC induction. EMA: in combination with a HMA for the treatment of adult patients with ND AML who are ineligible for IC.	+ Improved response and survival when combined with HMA or LDAC – Increased toxicity and myelosuppression compared to HMA only – Early bone marrow advised to adjust venetoclax treatment days in subsequent cycles – Dose modifications needed when combined with CYP3A4 inhibitors
CC-486 (ONUREG)	Oral azacitidine	FDA: continued treatment of adult patients with AML who achieved CR/CRi following IC induction and are not able to complete intensive curative therapy. EMA: maintenance therapy in adult patients with AML who achieved CR/CRi following IC induction therapy with or without consolidation treatment and who are not candidates for, including those who choose not to proceed to, HSCT.	+ Improved response and survival compared to wait-and-see – Gastrointestinal complaints common. Prophylaxis needed to increase treatment adherence

This review highlights the options for assessing fitness and will discuss clinical scenarios in elderly AML to guide individualized treatment for older patients with AML.

## Evaluating Fitness in the Older Patient

Many efforts have been undertaken to establish fitness and select suitable older AML patients for IC. Historically, older age has been seen as an important determinant of fitness and multiple large retrospective studies confirm age to be an independent prognostic factor for outcome in AML [14, 15, 16]. However, although age is clearly related to prognosis, factors associated with early treatment-related mortality (TRM) might be better fitness indicators. In that context, multicomponent models are more accurate than age alone [17]. Moreover, the elimination of age from these models only minimally affects their predictive accuracy, indicating that age is partly a surrogate for other covariates [17]. PS seems more predictive for early TRM than age [6, 17, 18] but there is a noticeable interaction. The likelihood of early death upon treatment with IC in patients with a PS of 3 increases significantly with age (0% in patients < 56 years vs. 82% in patients > 75 years), whereas the likelihood of comorbidities also increases with age [6]. Several risk score systems, like the hematopoietic stem cell transplantation-comorbidity index (HCT-CI), adult comorbidity evaluation 27 (ACE-27), and Charlson comorbidity index (CCI), have shown to be predictive of outcome in AML [5, 19, 20]. For example, a score of ≥ 3 on the HCT-CI in patients over 60 years is associated with an early mortality rate of 29% upon treatment with IC [19]. It is clear that comorbidities can limit treatment options and increase chances of toxicity but patients with well controlled comorbidities could still be candidates for IC. On the other hand, seemingly fit patients without relevant comorbidities can have considerable functional or cognitive impairment that is not necessarily directly noticeable during regular consultation. A structured geriatric assessment (GA) can help detect these impairments, initiate precautionary actions or early treatment, and discriminate fit from unfit patients. Pretreatment GA has predictive value for survival and other treatment-related outcomes of elderly patients with AML but can also be used during treatment, upon clinical improvement or decline, and guide care and decision-making [21, 22]. However, despite all tools available, the consensus and cut-off values to establish fitness remain rather arbitrary. Interestingly, clinical impression by the trained eye of physicians and nurses can estimate the patient’s clinical condition which is significantly associated with mortality and morbidity in hematology and oncology patients [23, 24]. We therefore use age, PS, comorbidity scores, and GA, all as tools to support the value of clinical impression of the treatment team to guide our treatment decisions.

## Clinical Scenarios

### The Fit Elderly Patient with Newly Diagnosed AML…

**Fig. 1:**
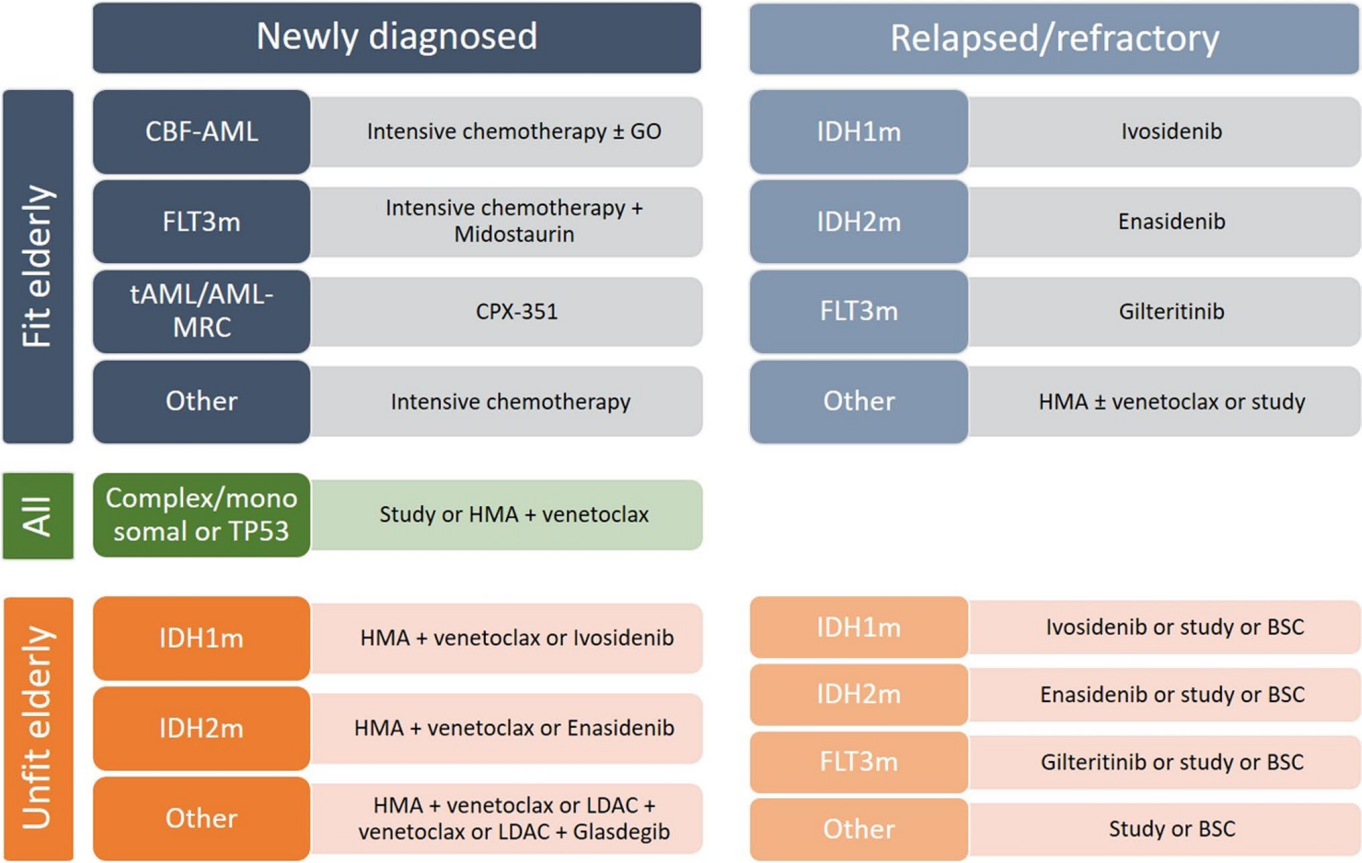
Purposed treatment algorithm for elderly AML patients. CBF-AML, core-binding factor acute myeloid leukemia; FLT3m, FMS-like tyrosine kinase mutated; tAML, therapy-related AML; AML-MRC, AML with myelodysplasia-related changes; GO, gemtuzumab ozogamicin; HMA, hypomethylating agent; IDH1m, isocitrate dehydrogenase 1 mutated; IDH2m, isocitrate dehydrogenase 2 mutated; LDAC, low-dose cytarabine; BSC, best supportive care.

To date, despite all the recent advances, the only potential curative treatment for patients with AML is IC induction followed by either post-remission intermediate or high dose cytarabine courses, autologous HCT consolidation, or treatment with an allogeneic stem cell transplant. IC in fit elderly patients is known to be feasible and provides a valuable option associated with a better long-term survival in older AML patients compared to HMA [25]. However, IC remains more toxic than HMA in older AML patients and is associated with increased early mortality in retrospective studies. Whether induction with decitabine can provide comparable remission rates with less toxicity and offers a better bridge to transplant than IC, is currently investigated in the randomized EORTC-1301 trial (NCT02172872). Although these results are awaited with great interest, the landscape of AML therapy has already moved on. The current standard for the treatment of elderly (> 75 years) and unfit AML is the combination of HMA and venetoclax. It has demonstrated excellent activity with favorable safety, even in frail patients [13••, 26]. Studies suggest that complete remission (CR) rates attained by these combinations may approach those of IC and it is therefore often hypothesized that HMA with venetoclax should also be the preferred frontline treatment in elderly AML patients (60–75 years) who are fit for IC treatment. This is supported by propensity matching-based studies that suggest equivalence between the two treatment modalities [27, 28]. However, these studies use historical data with a high potential of selection bias and they should therefore not be viewed as prove for equivalent effectiveness. A trial randomizing between IC and HMA with venetoclax is enrolling (NCT04801797). Until these trials prove otherwise, we suggest to reserve HMA with venetoclax only for patients who match the inclusion criteria of the VIALE-A study and use IC for fit elderly patients without comorbidities. An algorithm for the treatment of elderly AML patients is purposed in Fig. 1.

### … and a Favorable or Intermediate Risk Profile

The choice between “classical” 7 + 3, newer intensive treatment options, like CPX-351, or the addition of targeted drugs strongly depends on the ELN risk classification and the molecular profile. For patients with a good (CEBPa double mutant, CEBPa-bZIP, or core-binding factor AML) or intermediate-risk profile, without FLT3 or IDH1/2 mutations, standard 7 + 3 induction treatment results in CR in 60–70% of patients and is therefore a valid treatment option [29]. The addition of gemutuzumab ozogamicin (GO) can enhance the outcome for patients within this AML risk group [30–31]. GO is a humanized antibody-drug conjugate composed of a monoclonal antibody targeting CD33, covalently linked to the cytotoxic drug N-acetyl-γ-calicheamicin. It is approved by the FDA for the treatment of relapsed/refractory (R/R) and newly diagnosed (ND-AML) CD33-positive AML and has EMA approval for *de novo* CD33-positive patients. A recent meta-analysis showed an improved survival benefit, especially for patients aged < 70 years, with *de novo* AML, with positive expression of CD33, with *NPM1* mutation, without *FLT3-ITD* mutation, and with low-/intermediate-risk karyotypes [33]. Core-binding factor AML is another subtype that has shown improved survival with the use of GO in combination with IC although the toxicity of GO remains a concern especially in older patients. Reduction of the number of chemotherapy days when GO is combined with intensive treatment is advised for patients above the age of 60 [34]. Recently, GO was also investigated as a replacement for anthracycline (i.c. idarubicine) during induction of fit elderly patients in the randomized ALFA1401-Mylofrance 4 Study but this did not improve outcome and was associated with increased toxicity and a non-significant higher relapse incidence, shorter EFS, and shorter OS [35]. Although low- and intermediate-risk and CBF AML are less prevalent in elderly AML, and despite the concerns of toxicity, the addition of GO should be considered for fit elderly patients who present with these subtypes.

### … with Myelodysplasia-Related Changes or tAML

In contrast, a significantly greater proportion of elderly patients present with sAML. This subgroup, representing AML that arises form a preceding myeloid disease or tAML, is generally associated with a poor prognosis [36]. Recently, CPX-351 (Vyxeos) received FDA and EMA approval for the treatment of ND tAML or AML with myelodysplasia-related changes (AML-MRC). CPX-351 is a liposomal formulation of cytarabine and daunorubicine at a fixed 5:1 molar ratio with improved uptake in leukemic cells in vitro and results in a long half-life in human plasma [37, 38]. In fit elderly patients with ND-AML, age 60 to 75 years, CPX-351 has been shown to increase response rates compared to conventional IC, resp. 66.7% vs. 51.2% [39]. Moreover, CPX-351 improved mOS (9.56 vs. 5.95 months) in a comparable phase III study in elderly high-risk ND-AML and sAML. A recent update of this study showed twice as many patients alive at 3 and 5 years with CPX-351 than with IC, although patient numbers were low [40•]. Interestingly, patients who went on to HSCT especially seemed to benefit. Likely explanations are that CPX-351 induces deeper remissions with higher rates of measurable residual disease (MRD) negativity or leads to better clinical condition pre-transplant due to less toxicity [41, 42]. Importantly, CPX-351 is generally well tolerated and associated with low frequency of alopecia and gastrointestinal toxicity but is associated with prolonged time to neutrophil and platelet count recovery [42]. Based on current knowledge, we intent to use CPX-351 for fit elderly patients between 60 and 75 years with ND tAML or AML-MRC, especially when there is an intent to consolidate with a HSCT. For patients with tAML or AML-MRC who present with comorbidity, or are fit but do not want to proceed to HSCT, we tend to use low-intensity regimens.

### … with IDH1/2 Mutation

Patients who present with targetable mutations, like IDH1, IDH2, or FLT3, should preferably be treated in clinical trials investigating the addition of specific inhibitors combined with IC. Examples of such trials are the HOVON 150 (for IDH1/2 mutated AML, NCT03839771) and HOVON 156 (for FLT3 mutated AML, NCT04027309), in which fitness for intensive treatment, and not age itself, defines eligibility for these trials.

For IDH1/2 mutated AML, two inhibitors have been approved by the FDA. Ivosidenib is approved for ND-AML with an IDH1 mutation age 75 years or older, patients who are ineligible for IC, and R/R AML. Enasidenib is an IDH2 inhibitor and is approved for patients with R/R AML. Both inhibitors have shown variable results in the different treatment settings either as monotherapy or in combination with HMA [43–47••]. The studies supporting the use of these inhibitors in unfit patients are discussed in a different section of this paper. The combination of ivosidenib or enasidenib with IC induction has demonstrated encouraging responses and an acceptable safety profile in a phase I study [48]. Composite CR rates for ivosidenib and enasidenib in patients with de novo AML were 88% and 80%, respectively. The randomized phase III HOVON 150 trial investigating standard IC combined with either ivosidenib or enasidenib vs. placebo is currently ongoing.

### … with FLT3 Mutation

Over the last years, multiple FLT3 inhibitors have been developed and entered clinical trials at various disease stages, either as mono- or in combination therapy. The first-generation FLT3 inhibitors include midostaurin, sorafenib, and lestaurtinib. As a single agent, these inhibitors have shown limited efficacy and variable tolerability due to the adverse effects derived from their multikinase inhibitory activities [49-51]. However, combined with standard IC, midostaurin improved overall survival of ND patients with FLT3 mutated AML, as shown in the RATIFY trial where a 7% higher probability of survival after 48 months was seen than in patients treated with IC only [52]. Although this study did not enroll patients over 60 years, midostaurin is FDA and EMA approved for the treatment of ND FLT3 mutated AML of all ages who undergo IC induction. Interim-analyses of the AMLSG 16-10 trial, which included patients up to 70 years, reported this combination to be feasible and equally effective in elderly patients, despite the more prevalent dose reductions compared to younger patients [53].

Second-generation FLT3 inhibitors include gilteritinib, quizartinib, and crenolanib. These newer inhibitors are more potent and selective than first-generation inhibitors, improving their tolerability and efficacy [54]. This was demonstrated by two phase III trials in which single-agent gilteritinib (ADMIRAL) and quizartinib (QUANTUM-R) improved remission rates and mOS compared to high- and low-intensive types of salvage chemotherapy in R/R AML [55, 56•]. Whether second-generation inhibitors also improve outcome of ND patients when combined with IC, is currently under investigation in multiple phase III trials (NCT02668653, NCT04027309). We recommend to include fit elderly FLT3 mutated patients in these trials.

### … with Poor Risk Cytogenetics or TP53 Mutation

Elderly patients often present with unfavorable risk cytogenetics and poor-risk mutations. In particular, complex and monosomal karyotype and TP53 mutations confer very poor outcome, even in the context of HSCT [57, 58]. Attempts to improve outcomes in this subgroup thus far have been unsuccessful and also the addition of venetoclax failed to show clinical benefit in this context [59-61]. This raises the question whether to treat elderly TP53 mutated patients with intensive therapies that will reduce quality of life (QoL) without a reasonable chance of curation, or even, an acceptable improvement of disease free survival. We therefore tend to postpone treatment in elderly patients until cytogenetic and molecular results are available in order to make an informed treatment decision. Delaying treatment awaiting these results is not harmful in stable older AML patients [62-64]. In general, we do not commence treatment with IC in patients ≥ 65 years when extremely poor-risk features are found. Instead, we diverge to less toxic treatments with HMA and try to include these patients in specific clinical trials when available. Promising new therapies currently in clinical trials include the anti-CD47 antibody Magrolimab, the mutant p53 reactivator Eprenetapopt (APR-246), the dual-affinity molecule CD123 × CD3 Flotetuzumab, and the menin-inhibitor SNDX-5613 [65-69].

### … Eligible for Allogeneic Stem Cell Transplant

Although new therapies have improved overall survival of patients with AML, curation cannot be achieved without high dose chemotherapy or HSCT. HSCT has been shown to effectively reduce relapse risk in all AML risk groups but improvement of survival is predominantly seen in intermediate and high-risk patients [70, 71]. Although older patients more often present with high-risk disease and have a higher chance of relapse, the use of HSCT, until recently, was limited to patients under 60 years, as older patients were more likely to experience complications and have higher TRM rates. However, reduced-intensity conditioning (RIC) regimens, improved immunosuppressive strategies, and better supportive care have made HSCT now also feasible for elderly patients [72]. Consolidation with HSCT has increased over the last decades and is now performed in approximately 26% of all elderly (60–75 years) AML patients in the Netherlands [73]. The importance of bringing eligible elderly patients to HSCT is demonstrated by multiple retrospective studies showing that transplants can reduce relapse and improve long-term overall survival compared to non-transplant strategies, even despite the fact that transplant is associated with an increased risk of (early) TRM [74-76]. Moreover, prospective data from the ECOG-ACRIN study, in which patients between 60–73 years of age received a HSCT in CR1, show comparable results with an encouraging 4-year OS rate of 43%. Interestingly, the NRM rate in patients > 65 years was similar compared to patients ≤ 65 years [77]. Therefore, we attempt to bring all eligible elderly patients ≥ 65 years, who achieve complete remission on intensive or low-intensity induction treatments, to HSCT.

### … Non-eligible for Allogeneic Stem Cell Transplant

Unfortunately, HSCT is not feasible in all elderly patients. Patients who initially were fit at diagnosis can deteriorate due to treatment and complications and be deemed ineligible, or patients can decide to refrain from transplant based on personal preference. For these patients, low-intensity post-remission treatment to increase DFS and mOS is available. The Dutch-Belgian Hemato-Oncology Cooperative Group (HOVON) has investigated subcutaneous azacitidine maintenance after CR or CR with incomplete hematological recovery (CRi) after IC [78]. Maintenance with 50 mg/m^2^, for 5 days, every 4 weeks, was feasible and significantly improved DFS compared to observation, but OS did not differ between the groups.

CC-486 (ONUREG®) is an oral formulation of azacitidine. Compared to subcutaneously injected azacitidine, CC-486 has a different pharmacodynamic and pharmacokinetic profile, which allows for extended dosing schedules at lower dose, to prolong drug exposure with sustained epigenetic targeting [79]. CC-486 has received regulatory approval from the FDA and EMA as maintenance therapy for AML patients who have CR/CRi following IC with or without consolidation treatment and who are ineligible for, or choose not to proceed to, HSCT. Approval was based on results of the phase III, randomized, double-blind, placebo-controlled QUAZAR AML-001 trial (NCT01757535) [80••]. This trial investigated CC-486 maintenance versus placebo in patients aged ≥ 55 years with ND-AML, who were in remission after IC but not candidates for HSCT. Maintenance treatment with CC-486 resulted in improved mOS en mRFS compared to placebo (24.7 vs. 14.8 months and 10.2 vs. 4.8 months, respectively). Recently, updated survival analysis, with a median follow up of 51.7 months, showed a sustained, long-term OS benefit with CC-486 [81]. Long-term survival was associated with intermediate-risk cytogenetics and *NPM1* mutations at diagnosis and absence of detectable MRD after induction. Gastrointestinal complaints are common during treatment with CC-486 which can decrease QoL and lead to treatment discontinuation. It is suggested that patients develop progressive tolerance with continued treatment but counseling and prophylactic treatment with anti-emetics, proton-pump inhibitors, laxatives, and/or anti-motility agentsare advised to increase treatment adherence [82].

### The Unfit Elderly Patient with Newly Diagnosed AML…

For a large number of elderly patients, IC is deemed too toxic. For these patients, low-intensity treatments, such as HMA and low-dose cytarabine (LDAC), represent an effective alternative although the results remain unsatisfactory [12].

The addition of glasdegib, an oral smoothened inhibitor, in combination with LDAC was shown to improve response rates (CR/CRi 24.3% vs. 5.2%) and mOS (8.8 vs. 4.9 months) compared to LDAC alone [83]. Based on these results, glasdegib received FDA and EMA approval for the treatment of ND-AML in patients unfit for IC. However, response and survival rates are only modest and do not reach rates that are achieved with single-agent azacitidine.

There is a strong synergistic effect when venetoclax is combined with HMA [84]. The efficacy of the combination of venetoclax with azacitidine has been studied in the VIALE-A study [13••]. This randomized, double-blind, placebo-controlled, phase III trial showed improved OS in patients with ND-AML compared with azacitidine alone. Median OS increased from 9.6 to 14.7 months and the CR rate was significantly higher with the combination than with azacitidine alone, resp. 36.7% vs. 17.9%. The combination of venetoclax with decitabine has been investigated in several phase I/II studies and shows similar improvement in responses [26, 85, 86]. Combined with a 10-day decitabine regimen, an impressive CR/CRi rate of 84% in ND-AML could be achieved [87]. Improved responses and survival were also seen in patients treated with low-dose cytarabine when venetoclax was added, although responses overall were less than the combination with HMA and mOS (10.1 months) is more comparable to results obtained with HMA monotherapy [88]. Based on these studies, venetoclax has been granted approval by the FDA and EMA in combination with azacitidine, decitabine, or low-dose cytarabine for the treatment of ND-AML in adults ≥ 75 years, or who have comorbidities precluding IC induction.

In general, all combinations mentioned are well tolerated, but increased hematological toxicity and febrile neutropenia is common [86]. Compared to the treatment of CLL, tumor lysis syndrome with venetoclax in AML is infrequent. However, AML with NPM1 and/or IDH1/2 mutations may have increased risk due to their sensitivity. Outpatient treatment with venetoclax can be safe with careful monitoring but hospitalizations due to infectious complications are frequent awaiting hematologic recovery [89]. We therefore make an individualized decision and choose inpatient treatment for patients with high complication risk or poor social support networks.

### … with IDH1/2 Mutation

As mentioned above, IDH1/2 mutated AML is particularly sensitive to azacitidine-venetoclax. Analysis from the VIALE-A study shows CR/CRi of 66% vs. 9% and 86% vs. 11% for IDH1 and IDH2 mutated AML, respectively, when compared to azacitidine-placebo [90]. Treatment with azacitidine-venetoclax therefore is a valid treatment option for these patients.

Another possibility is ivosidenib, that is FDA approved for the treatment of unfit elderly patients with IDH1 mutated AML. The drug induces lower responses (CR/CRi/CRp 41.2%) compared to azacitidine+venetoclax; however, it is well tolerated and seems to have lower rates of cytopenia and infectious complications [44]. The AGILE trial showed that the combination of ivosidinib and azacitidine had a higher rate of CR (47.2% vs. 14.9%) and a significantly improved mOS (24.0 vs. 7.9 months) compared to single-agent azacitidine treated patients [47••]. Notably, the combination was well tolerated and showed a reduced frequency of infections and a trend towards improved QoL and symptom burden.

In elderly IDH2 mutated ND-AML patients, treatment with enasidenib resulted in a CR rate of 18% [91]. The combination of enasidenib and azacitidine was studied in a large randomized phase II trial (AG221-AML-005) and significantly improved response (CR/CRi 57% vs. 18%) and duration of response (DOR) (24.1 vs. 9.9 months) compared to azacitidine monotherapy. Despite this improvement, the study failed to demonstrate a survival benefit, possibly due to post-study use of enasidenib in patients progressing after azacitidine [46].

Although ivosidenib and enasidenib are well tolerated, both are associated with differentiation syndrome. This potentially fatal adverse reaction is seen in around 19% of patients treated with IDH inhibitors and early recognition and treatment is critical to prevent severe complications and mortality [92, 93].

As IDH inhibitors are not globally available and responses are excellent, we tend to treat our IDH1/2 mutated unfit elderly patients with azacitidine-venetoclax. When available, monotherapy ivosidenib could be considered for the frailest patients or for those who do not want repeated hospital visits for azacitidine injections. Whether triplet regimens with azacitidine, venetoclax, and an IDH inhibitor induce even better responses is currently under investigation (NCT03471260) [94, 95].

### … with FLT3 Mutation

Currently, the standard treatment of unfit elderly patients with a FLT3 mutation is azacitidine-venetoclax. This combination demonstrated a favorable response rate compared to azacitidine monotherapy (CR/CRi 67 vs. 36%), and longer mOS (12.5 months vs. 8.6 months) [13••, 96]. The addition of gilteritinib to azacitidine has been investigated in the LACEWING trial [97]. This randomized phase III study failed to demonstrate a survival benefit despite a significantly improved composite CR (CRc) rate compared to azacitidine monotherapy, 58.1% vs. 26.5%, respectively. The higher percentage of patients with an ECOG > 2 in the combination arm and the more frequent use of post-study TKI treatment in the control arm could have confounded the OS findings.

Whether triplet therapy, combining a FLT3 inhibitor, venetoclax, and a HMA could improve outcome is currently investigated in clinical trials. Maiti et al. reported on 12 elderly ND-AML patients treated with 10-day decitabine combined with venetoclax and various FLT3 inhibitors (gilteritinib, sorafinib, or midostaurin) [98]. The triplet demonstrated a high CRc rate of 92% and an 18-month progression free survival of 59%. Although the combination was well tolerated, triplet regimens seem to be more myelotoxic. Updated reports suggest dose modifications for gilteritinib and early bone marrow evaluation to evaluate marrow ablation and subsequent withholding of venetoclax in order to allow for neutrophil recovery.

### … and a Poor Performance Status

Although low-intensity treatment can significantly improve survival compared to supportive care, not all elderly patients are able, or want, to undergo treatment. Prognosis is particularly poor in the eldest elderly (≥ 80 years) with a poor PS and in these patients, BSC can be a valuable option. Importantly, active AML can contribute to poor PS and, in some patients, treatment may improve performance and enhance the patient’s ability to tolerate and benefit from subsequent treatment. It is therefore important to carefully distinguish chronic comorbidities from transient, and potentially improvable, AML related complications. Hydroxyurea, transfusion, and antimicrobial prophylaxis can be applied to patients who are only candidates for BSC. Importantly, specialty palliative care is recommended to improve QoL, psychological distress, and end of life care [99].

## The Elderly Patient with Relapsed AML

Unfortunately, recurrence of AML after CR is frequently seen in elderly AML and prognosis is then extremely poor with an mOS of at highest 6 months [100, 101]. Management of these patients is highly dependent on the clinical context, disease biology, and presence of targetable mutations, and therefore, treatment strategies range from reinduction with salvage chemotherapy to BSC. Treatment with curative intent may be attempted for the “younger elderly” patients who have the possibility to undergo HSCT or donor lymphocyte infusion after achievement of a second CR. Salvage regimens with IC should only be considered for exceptionally fit patients with late relapses (> 1 year) without poor-risk features and targetable mutations. For patients with IDH1/2 or FLT3 mutations, targeted treatment with ivosidenib, enasidenib, or gilteritinib has shown to be effective and less toxic than IC. When targetable mutations are absent, less intensive treatment with HMA, when possible combined with venetoclax, is the preferred option for less-fit patients who have not been pretreated with these agents before [102, 103]. However, for most patients, especially the eldest elderly or patients with poor-risk features and/or early relapse, treatment is mainly palliative. Enrollment in clinical trials is encouraged for all patients as new therapies and combinations are under investigation that are desperately needed to improve outcome for these patients.

## Conclusion

Treatment of the elderly patient with AML is challenging due to the heterogeneity between aging patients and their diverse disease biology. Outcome remains poor, especially for those who are not candidates for HSCT. Introduction of new drugs has now increased treatment options for both fit and unfit elderly patients. Especially in elderly patients, factors as age, PS, comorbidity, cognitive and physical functioning, social network, and diseases biology must be weighed against the various treatment options with different response rates and side effects. Individualized and tailored medicine is therefore needed to select the best fitting therapy for each patient.

## Abbreviations

ACE-27: adult comorbidity evaluation 27
AML: acute myeloid leukemia
BSC: best supportive care
CBF: *c*ore-binding factor
CCI: Charlson comorbidity index
CR: complete remission
CRc: composite complete remission
CRi: complete remission with incomplete hematological recovery
DFS: disease-free survival
FLT3: FMS-like tyrosine kinase 3
GA: geriatric assessment
GO: gemtuzumab ozogamicin
HCT-CI: hematopoietic stem cell transplantation-comorbidity index
HMA: hypomethylating agents
HOVON: the Dutch-Belgian Hemato-Oncology Cooperative Group
HSCT: allogeneic hematopoietic stem cell transplantation
IC: intensive chemotherapy
IDH: isocitrate dehydrogenase
LDAC: low-dose cytarabine
mOS: median overall survival
MRC: myelodysplasia-related changes
MRD: measurable residual disease
*ND*: newly diagnosed
NRM: non-relapse mortality
OS: overall survival
PS: performance status
QoL: quality of life
R/R: relapsed/refractory
RFS: relapse-free survival
RIC: reduced-intensity conditioning
sAML: secondary AML
tAML: therapy-related AML
TRM: treatment-related mortality
